# Tuberous sclerosis presenting along with autosomal dominant polycystic kidney disease (ADPKD): A rare presentation

**DOI:** 10.6026/973206300211705

**Published:** 2025-05-31

**Authors:** Pushpendra Singh Sengar, Amit Saxena, Anurag Jain

**Affiliations:** 1 Department of Medicine, Bundelkhand Medical College and Hospital, Sagar, Madhya Pradesh, India

**Keywords:** Tuberous sclerosis complex (TSC), autosomal dominant polycystic kidney disease (ADPKD), TSC2-PKD1 contiguous gene syndrome, renal manifestation, genetic syndromes, neurocutaneous syndromes, renal cysts

## Abstract

Tuberous sclerosis complex (TSC) and autosomal dominant polycystic kidney disease (ADPKD) are two different genetic diseases. Although these two diseases are associated very rarely, the association is well recognized. This occurs due to a large deletion involving both PKD-1 and TSC-2 genes on chromosome 16. This is also known as TSC-2/PKD-1 contiguous gene syndrome. We present a case of a 19-year-old male, presenting with hematuria and tuberous sclerosis phenotype whose USG abdomen and CT scan of head and abdomen revealed bilateral multiple renal cysts and sub-ependymal nodules suggestive of having Tuberous sclerosis along with ADPKD.

## Background:

Tuberous sclerosis complex (TSC) is a rare autosomal dominant disorder with an estimated incidence of 1 in 6,000 to 1 in 11,000 live births. TSC is caused by mutations in either the TSC1 or TSC2 genes, with TSC1 encoding hamartin located on chromosome 9q34 and TSC2 encoding tuberin located on chromosome 16p13. These mutations disrupt cellular growth regulation, leading to the development of benign tumors (hamartomas) in multiple organs, including the brain, skin, heart, lungs and kidneys. Renal manifestations are common in TSC, including angiomyolipomas (present in up to 85% of patients), renal cysts (44.8%) and rarely, renal cell carcinoma. These complications can lead to renal dysfunction and hypertension, which require careful monitoring and management.

## Autosomal dominant polycystic kidney disease (ADPKD):

ADPKD is a systemic genetic disorder that leads to the formation of numerous fluid-filled cysts in the kidneys. It is most commonly caused by mutations in the PKD1 gene, located on chromosome 16p13.3, or the PKD2 gene on chromosome 4q21-q23. The disorder presents with symptoms such as hypertension, abdominal pain, hematuria, urinary tract infections and the gradual development of renal failure [1, 2]. In some cases, ADPKD coexists with TSC, resulting in a rare contiguous gene syndrome. This occurs due to deletions on chromosome 16 that involve both the TSC2 and PKD1 genes. Such patients are at increased risk for early-onset renal failure and they may experience accelerated renal cyst formation and a higher risk of renal malignancies [3, 4]. Although the co-occurrence of TSC and ADPKD is extremely rare, it is clinically significant and poses unique challenges for diagnosis and management. This case report details the rare association of TSC and ADPKD in a 19-year-old male patient, diagnosed after presenting with hematuria [5, 6].

## Case presentation:

A 19-year-old male patient presented to our clinic with a history of hematuria for the past two months. The patient had no prior significant medical history but had a family history of hypertension and renal disease. Upon examination, he did not show any signs of systemic involvement from TSC, such as seizures, cognitive impairment, or cutaneous lesions. Initial imaging studies revealed multiple renal cysts and further investigation confirmed the presence of angiomyolipomas in both kidneys. Genetic testing identified a mutation in the TSC2 gene, confirming a diagnosis of tuberous sclerosis complex. Additionally, renal ultrasonography and CT scan findings were consistent with autosomal dominant polycystic kidney disease (ADPKD), with bilateral cyst formation and no signs of malignancy. Thus, the patient was diagnosed with the rare co-occurrence of TSC and ADPKD, a combination found in less than 2% of TSC cases.

## Case report:

[1] A 19 year old male who presented with complaint of hematuria and fever was admitted in the department of surgery and was transferred to the department of medicine as his USG abdomen was suggestive of cystic disease. (USG findings - B/L kidneys enlarged with multiple cysts of variable size scattered diffusely with cyst size being 37*34mm s/o ADPKD). His medical history was not significant. The patient had a positive family history for similar lesions on the face of his mother and sister.

[2] On physical examination, the patient was found to have multiple angiofibromas over the nasolabial folds, hypomelanotic macules and confetti skin lesions over the back which was present since childhood raising the suspicion of tuberous sclerosis. Due to the presence of 2 major features, we diagnosed the case as tuberous sclerosis.

[3] On examination Patient's vitals were stable, Neurological, Cardiac, respiratory and per abdomen examination were within the normal limits.

## Investigations:

His laboratory investigations were significant for:

Elevated serum urea = 96mg/dl

Serum creatinine = 3.5mg/dl

Serum uric acid = 8.0mg/dl

Urine c/s not suggestive of any growth

Urine r/m:

RBC count =40-50/hpf

Pus cells =20-25/hpf

s/o AKI

USG Abdomen - B/L kidneys enlarged with multiple cyst of variable size scattered diffusely with cyst size being 37*34mm and distorted architecture with RT kidney measuring 15x8cm and Lt Kidney 15x7cm s/o ADPKD. CT HEAD-coarse calcified foci seen along the right subependymal region of the lateral ventricles along with patchy hypodense areas seen along the right frontal, left frontal, left posterior temporal and left high parietal lobe in the subcortical and cortical location. CT ABDOMEN- Bilateral kidney appears enlarged with multiple well defined cystic lesions seen scattered diffusely in both kidney, replacing the parenchyma, cysts size.

Right-29x32mm,

Left-36x43mm

Pure Tone Audiometry- No SNHL

Fever and hematuria had subsided on symptomatic treatment for 3 days consisting of IV fluids, antipyretics and antibiotics.

## Discussion:

## Renal involvement in tuberous sclerosis complex (TSC):

Renal manifestations are commonly seen in tuberous sclerosis complex (TSC), with the most frequent being angiomyolipomas, renal cystic disease and, less often, renal cell carcinoma. Angiomyolipomas affect approximately 85.4% of TSC patients, while cystic kidney disease occurs in 44.8% and renal cell carcinoma in 4.2%. Although renal failure is a rare complication in TSC (occurring in about 1% of patients), it remains the second most common cause of mortality in TSC patients, after central nervous system complications. The frequency of angiomyolipomas and renal cysts tends to be higher when the TSC2 gene is involved, compared to TSC1 mutations. Worldwide, more than thirty cases of TSC associated with polycystic kidney disease (PKD) have been reported.

## Types of cystic disease in tuberous sclerosis:

Cystic disease in TSC typically presents in two forms:

[1] Simple renal cysts: The most common type, characterized by either single or multiple small cysts. These are generally asymptomatic but exhibit histological similarities to simple renal cysts.

[2] Polycystic kidney disease (ADPKD) manifestation: In rarer cases, TSC is accompanied by ADPKD, which is linked to worse renal prognosis due to the rapid progression of cyst formation and kidney damage.

## Adult polycystic kidney disease (ADPKD):

ADPKD is a separate genetic condition from TSC, resulting from mutations in the PKD1 gene on chromosome 16 (responsible for 85% of cases) or the PKD2 gene on chromosome 4 (found in 15% of cases). In ADPKD, renal cysts gradually increase in size and number, leading to progressive kidney dysfunction. Molecular studies have shown that the TSC2 and PKD1 genes are located close to each other on chromosome 16, which is important in understanding the association between TSC and ADPKD [7, 8].

## TSC-2/PKD-1 contiguous gene syndrome:

A rare genetic disorder, known as TSC-2/PKD-1 contiguous gene syndrome, occurs when a large deletion on chromosome 16 affects both the TSC2 and PKD1 genes. This syndrome is seen in around 2% of TSC patients. The deletion of both genes leads to the development of severe renal cystic disease, which shares similar radiological and morphological features with ADPKD [9]. In a study by Sampson *et al.*, constitutional deletions of TSC2 and PKD1 genes were linked to severe renal cystic disease, which resembled the clinical presentation of ADPKD. Patients with ADPKD and TSC often present with symptoms like abdominal distension, secondary hypertension and in some cases, hematuria due to the rupture of renal cysts [10]. The TSC2/PKD1 contiguous gene syndrome is a rare genetic disorder caused by a large deletion on chromosome 16p13.3 that simultaneously affects the TSC2 and PKD1 genes. This results in the co-occurrence of Tuberous Sclerosis Complex (TSC) and Autosomal Dominant Polycystic Kidney Disease (ADPKD), two distinct conditions with overlapping but severe clinical manifestations. Patients typically present with features of TSC, such as cortical tubers and skin lesions, alongside polycystic kidney disease characterized by multiple renal cysts, early-onset hypertension, and rapid progression to end-stage renal disease. Case reports, including one of a 30-year-old man presenting with acute loin pain, highlight the diagnostic challenges and serious renal outcomes associated with this syndrome [10- 11]. Genetic studies, such as the one by Brook-Carter *et al.*, have confirmed that these large deletions lead to early and severe renal involvement, often appearing in infancy or early adulthood. Due to the high risk of renal failure and potential for malignancy, early recognition of this syndrome is essential for timely intervention and management [4].

## Conclusion:

The coexistence of tuberous sclerosis complex and autosomal dominant polycystic kidney disease due to a contiguous gene deletion involving TSC2 and PKD1 represents a rare but significant genetic syndrome. Early recognition of this association is crucial, as it has implications for diagnosis, management, genetic counselling and prognosis. This case underscores the importance of thorough clinical and radiological evaluation in patients with features of TSC and unexplained renal cystic disease, particularly at a young age.
